# Amyloid precursor protein and amyloid precursor-like protein 2 in cancer

**DOI:** 10.18632/oncotarget.7103

**Published:** 2016-01-31

**Authors:** Poomy Pandey, Bailee Sliker, Haley L. Peters, Amit Tuli, Jonathan Herskovitz, Kaitlin Smits, Abhilasha Purohit, Rakesh K. Singh, Jixin Dong, Surinder K. Batra, Donald W. Coulter, Joyce C. Solheim

**Affiliations:** ^1^ Eppley Institute for Research in Cancer and Allied Diseases, University of Nebraska Medical Center, Omaha, NE, USA; ^2^ Department of Biochemistry and Molecular Biology, University of Nebraska Medical Center, Omaha, NE, USA; ^3^ Department of Pathology and Microbiology, University of Nebraska Medical Center, Omaha, NE, USA; ^4^ Fred and Pamela Buffett Cancer Center, University of Nebraska Medical Center, Omaha, NE, USA; ^5^ Department of Pediatrics, University of Nebraska Medical Center, Omaha, NE, USA; ^6^ Department of Stem Cell Transplantation and Cellular Therapy, University of Texas MD Anderson Cancer Center, Houston, TX, USA; ^7^ Wellcome Trust/DBT India Alliance Intermediate Fellow, CSIR-Institute of Microbial Technology, Chandigarh, India

**Keywords:** amyloid precursor protein, amyloid precursor-like protein 2, cancer, growth, migration

## Abstract

Amyloid precursor protein (APP) and its family members amyloid precursor-like protein 1 (APLP1) and amyloid precursor-like protein 2 (APLP2) are type 1 transmembrane glycoproteins that are highly conserved across species. The transcriptional regulation of APP and APLP2 is similar but not identical, and the cleavage of both proteins is regulated by phosphorylation. APP has been implicated in Alzheimer's disease causation, and in addition to its importance in neurology, APP is deregulated in cancer cells. APLP2 is likewise overexpressed in cancer cells, and APLP2 and APP are linked to increased tumor cell proliferation, migration, and invasion. In this present review, we discuss the unfolding account of these APP family members’ roles in cancer progression and metastasis.

## INTRODUCTION

**Figure 1:**
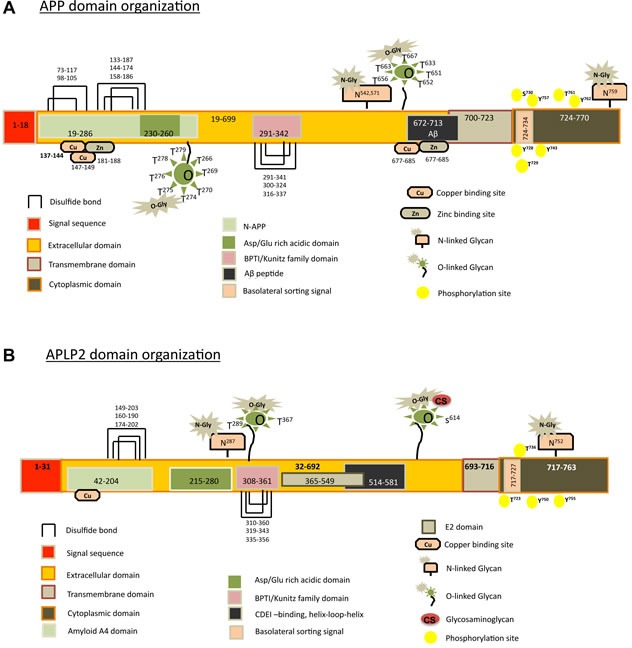
Graphical representations of the domains and sub-domains for APP and APLP2 are shown, along with disulfide bonds and predicted post-translational modifications **A.** For APP, the 770 amino acid isoform (UniProt accession number P05067) is displayed. **B.** For APLP2, the 763 amino acid isoform (UniProt accession number Q06481) is shown. N-glycan prediction was done with the NetNGlyc 1.0 Server (http://www.cbs.dtu.dk/services/NetNGlyc/). O-glycan prediction was performed by NetOGlyc 3.1 Server (http://www.cbs.dtu.dk/services/NetOGlyc/). Phosphorylated residues were predicted with PhosphoSite (http://www.phosphosite.org/homeAction.do). For some isoforms of APLP2, but not of APP, chondroitin sulfate (CS) glycosaminoglycan modification occurs at Serine 614.

Amyloid precursor protein (APP) is an evolutionarily conserved protein with two homologues in mammals: amyloid precursor-like protein 1 (APLP1) and amyloid precursor-like protein 2 (APLP2) [1, 2]. APLP1 was identified as the first homologue of APP [3, 4], and APLP2 (also known as YWK-II) was subsequently identified as an APP homologue [5-8]. All of these three proteins share high sequence homology and conserved domain structure. Each has an extracellular domain, a transmembrane-spanning domain and a ∼50 amino acid-long cytoplasmic tail domain (shown for APP and APLP2 in Figure 1A, 1B) [9,10]. APP and APLP2 are broadly expressed, while APLP1 expression is restricted to neural tissue [5, 6, 11, 12]. As described in detail below, APP and APLP2 are overexpressed in many cancers. APP and/or APLP2 have been described as having notable functions in many cancers, such as cancers of the prostate, breast, colon, thyroid, lung, nasopharynx, and gastrointestinal tract (for a more complete list, see Table 1). Both APP and APLP2 have been linked to characteristics of cancer cells such as abnormal growth, migration, and invasion (Table 1).

**Table 1:** Expression and role of APP and APLP2 in multiple cancer cell types

**Cancer**	**APP Expression and Effects**		**APLP2 Expression and Effects**	
Acute myeloid leukemia	↑	APP increases cell migration, as well as extramedullary infiltration due to matrix metalloproteinase-2 [67].	-	-
Breast	↑	APP increases cell growth, motility, survival, and phosphorylation of AKT pathway proteins [63, 64].	-	APLP2 is differentially spliced in breast cancer cell lines and human mammary epithelial cells [47].
Colon	↑	APP increases phosphorylation of ERK pathway proteins and increases proliferation [62, 68].	↑	APLP2 increases proliferation [69].
EBV-negative Burkitt's lymphoma	↑	APP causes rapid proliferation of Epstein-Barr virus-negative Burkitt's lymphoma cells [70].	-	-
Ewing's sarcoma	-	-	↑	APLP2 interferes with radiation-induced apoptosis and reduces MHC class I expression; APLP2 is increased in immune-evasive Ewing's sarcoma cells [71, 72].
Gastrointes-tinal neuro-endocrine	↑	APP is expressed in intestinal carcinoids, and is colocalized partly with markers of microvesicles and early endosomes [73].	↑	APLP2 is expressed in intestinal carcinoids, and is colocalized partly with markers of microvesicles and early endosomes [73].
Lung	↑	APP increases proliferation and causes cell size abnormalities [74].	↓	APLP2 expression is decreased in lung neuroendocrine tumors, though the consequences are not well understood [73].
Melanoma	↑	Perinuclear APP staining and soluble APP secretion are increased. APP facilitates proliferation, and its knockdown induces differentiation [75].	-	APLP2 decreases HLA class I surface expression on MDA-MB435S cells (formerly classified as breast cancer cells but recently classified as melanoma cells) [76].
Naso-pharynx	↑	APP increases cell growth and migration, and there is EGFR-mediated upregulation of soluble APP production [77].	-	-
Oral	↑	Upregulation of AP2α and positive correlation between APP and AP2α in tumor tissue [78].	-	-
Pancreas	↑	APP increases proliferation [42, 60, 68].	↑	APLP2 increases migration, proliferation, invasion, and metastasis [42, 61].
Prostate	↑	APP increases proliferation and migration, modulates levels of metalloproteinase and EMT-related proteins, and downregulates MAP kinase phosphatase and Notch signaling pathways [65].	-	-
Testicular germ cell	↑	APP expression was detected in ∼39% of nonseminomatous germ cell tumors (NSGC), and APP was associated with venous invasion [79].	↑	APLP2 is expressed in testicular germ cell tumor tissue [80].
Thyroid	↑	APP staining is increased in tumor tissue, and is associated with bigger tumor size, extracapsular invasion, and spread to the lymph nodes [81].	-	-

## APP AND APLP2 GENES AND PROTEIN STRUCTURES

The APP family members are encoded by separate multi-exon genes located on three different chromosomes [13-15]. The human APP gene is positioned at chromosome 21 (specifically, at band 21q21), and the human APLP2 gene is found at 11q24 [16, 17]. Although APP and APLP2 are related genetically, it has been observed that they are transcriptionally divergent, and there are unique sequence motifs in each gene that suggest specialized, non-overlapping functions [18].

The extracellular domain of APP contains two disulfide knots (three overlapping disulfide bonds) that resemble the disulfide knots within growth factors [19]. The cysteine residues used to form the disulfide knots of APP are conserved in APLP2, which by similarity is proposed to have two disulfide knots as well (Figure 1A, 1B). In the extracellular portion of APP and APLP2 are several smaller domains, including a bovine pancreatic trypsin inhibitor (BPTI)/Kunitz protease inhibitor domain, and a domain in which aspartic and glutamic acid residues are very abundant (called the Asp-Glu-rich domain) (Figure 1A, 1B) [9, 20].

The Kunitz protease inhibitor domain in the extracellular region of APP and APLP2 (Figure 1A, 1B) inhibits multiple proteases (such as trypsin, chymotrypsin, plasmin, plasmin, and kallikrein enzymes) with varying efficiencies [21]. Another protease inhibited by the Kunitz protease inhibitor domain (as part of the cleaved, secreted APP ectodomain) is coagulation factor XIa [22]. Notably, mesotrypsin produced by tumor cells acts to cleave the Kunitz protease inhibitor domain of the soluble, secreted APP ectodomain, which likely contributes to the procoagulant character of the tumor microenvironment [23].

At the positions indicated in Figure 1, the extracellular domains of APLP2 and APP bind copper or zinc ions [9, 24-27]. Recently, it was shown that the presence of copper increases the expression of APP in prostate cancer cells [28]. Furthermore, this same study revealed that the copper-binding region of the APP 770 splice variant (in conjunction with tyrosine residues in the APP intracellular domain) reduces copper-mediated inhibition of prostate cancer cell growth [28].

## CLEAVAGE OF APP FAMILY MEMBERS

**Figure 2:**
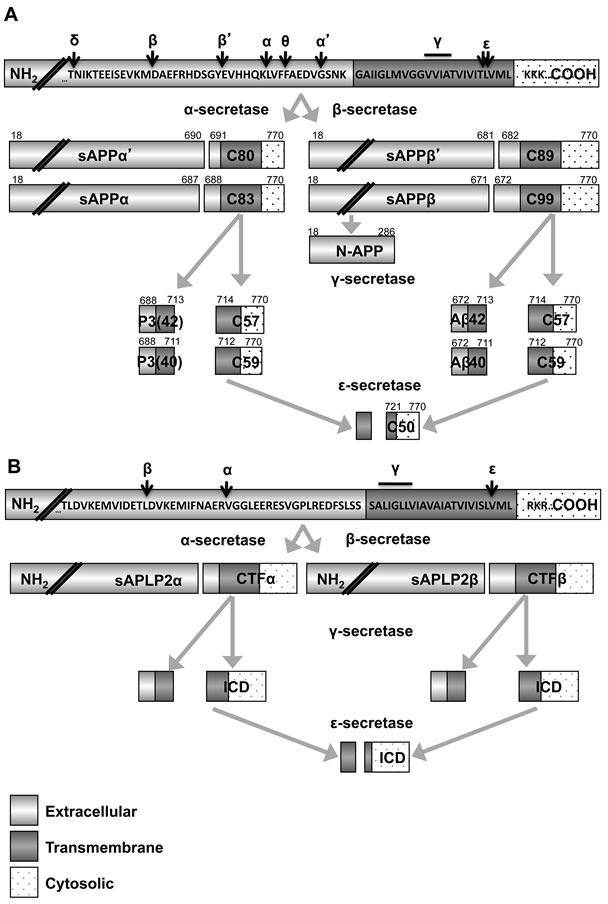
Secretase processing of APP and APLP2 generates many fragments Fragments of APP **A.** and APLP2 **B.** generated following cleavage by several secretase enzymes are shown. Amino acids in canonical APP and APLP2 are provided. Double forward slashes are used to denote truncated sequences. The N-terminal ends are indicated with NH_2_ and the carboxyl ends are indicated with COOH. **A.** Beta secretase 1 and 2 (BACE1 and BACE2) can cleave at site β, while the alternate cleavage site for BACE1 is site β’ and the alternative cleavage site for BACE2 is site θ. Two α-secretase cleavage sites have been described, α and α’. Sites γ and ε are cleaved by γ-secretase enzymes. Fragments of APP generated by various cleavage sites are provided with their nomenclature (text inside rectangles) and the residues forming the various fragments (superscript text above rectangles). The C-terminal fragments (CTF) of APP are C99, C89, C83 and C80 and the intracellular domains (ICDs) of APP are C59, C57 and C50. **B.** The BACE1 (β), ADAM10 (α) and γ-secretase cleavage sites (γ) have been determined for APLP2. APLP2 C-terminal fragments are distinguished by the secretase responsible for their formation. Intracellular domains of APLP2 are denoted as ICDs.

Of the three APP family members, APP is the most studied, notorious for its cleavage generating the β amyloid peptide (Figure 1A) that contributes to Alzheimer's disease [29-31]. Each of the APP family members (APP, APLP1, and APLP2) undergoes cleavage by secretases that release a large extracellular domain and produce smaller C-terminal fragments (Figure 2) [32-33]. APP and APLP2 cleavage is regulated by the hormone insulin-like growth factor 1 (IGF-1) [34-37]. IGF-1 has a known role in cancer progression, and multiple studies have explored the impact of inhibiting IGF-1 receptor signaling as a potential therapeutic approach for cancers, including neuroblastoma [38-41]. Some reports suggest that IGF-1 increases α-secretase cleavage of APP and APLP2, which would presumably result in down-regulation of β amyloid production [34, 36]. However, other reports indicate that IGF-1 actually promotes the generation of β amyloid [35], and therefore additional studies are needed to fully discern the effect of IGF-1 on APP and APLP2 processing and function in cancer.

In our studies, we have examined the effect of blocking beta-secretase activity on the viability of pancreatic cancer cells [42]. Many chemical inhibitors of beta-secretase have been developed, and some have already shown safety and efficacy in clinical trials for Alzheimer's disease patients [43-46]. We incubated pancreatic cancer cells with inhibitors of the beta-secretase enzyme, and observed a reduction in APLP2 C-terminal fragments in the cells. Furthermore, we demonstrated that treatment of the pancreatic cancer cells with beta-secretase inhibitors decreased the growth and viability of the cells. A non-transformed pancreatic cell line was included as a control, and the beta-secretase inhibitors did not diminish the growth or survival of the non-transformed cell line. Our results suggest that although no chemical inhibitors have been designed with the specific goal of targeting APLP2, existing beta-secretase inhibitors that have been made to target APP for the treatment of Alzheimer's disease may potentially be repurposed to target APLP2.

## APLP2 SPLICING

**Figure 3:**
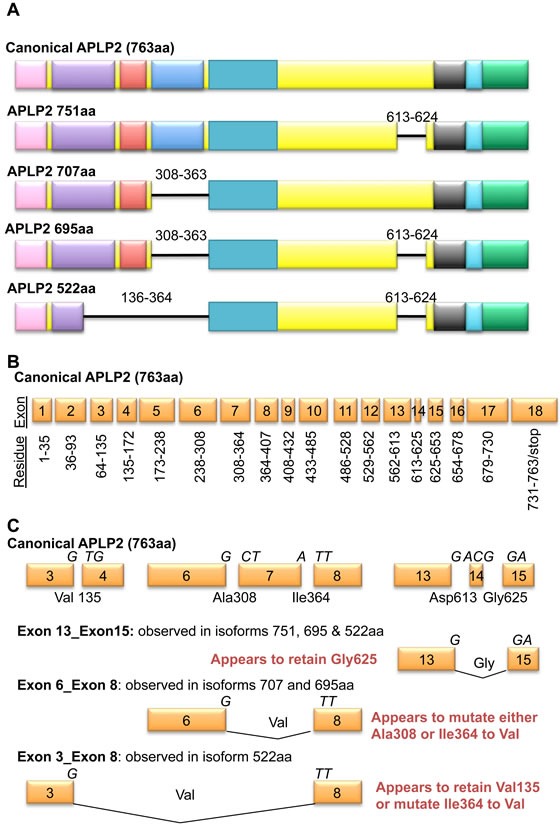
Isoforms of APLP2 arise through alternate splicing **A.** Reported isoforms of APLP2 are shown with numbers denoting residues not encoded in smaller isoforms. **B.** The 18 exons of APLP2 are displayed alongside the residues that they encode. **C.** Exon junctions in the canonical APLP2 sequence (top row) and known splicing sites (bottom three rows) are depicted. Letters above exons indicate the nucleic acid codes, and residues and their location within the canonical APLP2 isoform are provided.

In APLP2 (and in APP, as will be described below), splicing leads to diversity and to specialized functions influential in cancer. Several isoforms of APLP2 arise from splicing (Figure 3) [14, 20, 21]. The canonical isoform of human APLP2 is 763 residues long and ∼110 kDa in molecular mass, and it utilizes all 18 exons. Alternative APLP2 isoforms of 751, 707, 695, and 522 amino acids in length have been reported, and the first four of these isoforms arise from exclusion of exons 7 and/or 14. Omission of exon 7 removes the Kunitz protease inhibitor domain, and the frequency with which this APLP2 exon is excluded was found to vary in a comparison of two human breast cancer lines (MCF7 and MDA-MD-231) and human mammary epithelial cells [47].

Only APLP2 isoforms that exclude exon 14 are modified by chondroitin sulfate glycosaminoglycan modification on Ser614 (Figures 1 and 3) [48, 49]. Among the pancreatic cancer cell lines that we examined, 4 out of 5 expressed high levels of endogenous chondroitin sulfate glycosaminoglycan-modified APLP2 [42], which suggests that there may be preferential expression in pancreatic cancer cells of APLP2 isoforms lacking exon 14. Since transfected cells expressing various APLP2 isoforms had an increased migratory response when the isoform that was overexpressed was APLP2-751 (which bears the chondroitin sulfate glycosaminoglycan modification) [50], it is possible that the presence of the chondroitin sulfate glycosaminoglycan modification may also increase the migratory tendencies of pancreatic cancer cells.

## APP SPLICING

**Figure 4:**
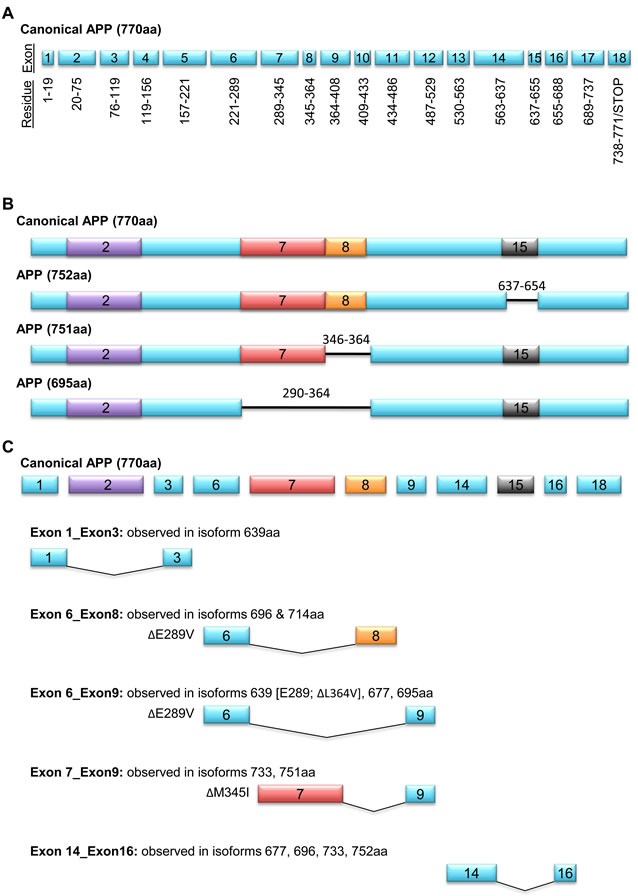
APP has alternatively spliced isoforms **A.** 18 exons encode the canonical 770-amino acid APP uncleaved glycoprotein, with several overlapping residues resulting from ligated mRNA of different exons forming a single codon. **B.** Diagrams of primary isoforms APP 751, APP 695, and leukocyte (L)-APP 752, with excised residues denoted by black horizontal lines. Excised exons are differentially colored: heparin-binding domain in exon 2 (purple), Kunitz-type protease inhibitor in exon 7 (red), OX-2 antigen sequence in exon 8 (orange), and amyloid beta processing sequence (grey). The deletion of the amyloid beta processing sequence enables the attachment of chondroitin sulfate glycosaminoglycan. **C.** Alternative splicing events and amino acid substitutions in 8 APP isovariants.

As is the case with APLP2, splicing also leads to generation of APP variants that differ in molecular mass. Alternative splicing of APP RNA gives rise to at least 9 translated isoforms, with variable expression across normal and disease states. The largest isoform, which contains the sequences encoded by all 18 exons, is 770 amino acids in length (Figure 4A). Aside from the canonical APP-770, the other primary APP isoforms contain 751 or 695 amino acid residues (Figure 4B). Some APP splice variants are restricted in localization. For example, APP-751 and APP-695 are abundantly present in mammalian brain [51-53], and APP 752, 733, 696, and 677 are found in astrocytes and leukocytes [54]. Upon mitogenic stimulation with phytohemagglutinin, T cells secrete APP-733 [55]. In the various APP isoforms, the spliced regions include a group of N-terminal residues near a heparin-binding domain (exon 2), the Kunitz-type protease inhibitor domain (exon 7), an OX-2 antigen domain (exon 8), and the amyloid beta processing sequence (exon 15) (Figure 4C).

Several studies have demonstrated that in cancer there is upregulation of the 751-amino acid APP isoform, which lacks the OX-2 antigenic domain encoded by exon 8 [56-58]. High throughput reverse transcriptase-polymerase chain reaction screens that profiled the expression of >600 cancer-related genes revealed the APP-751 splice variant in primary epithelial ovarian tumors and primary breast tumors, but not in corresponding tissues from normal donors, suggesting that the production of APP-751 is cancer-specific rather than simply characteristic of the tissue of origin [56-58]. The high degree of APP exon 8 excision in breast tumor versus normal tissue (p<10^−5^) prompted its inclusion in a 12-marker APP splice variant panel that correctly identified 33 of 35 (96%) of tumor samples in a blinded validation assay. Likewise, quantitative microarray followed by reverse transcriptase-polymerase chain reaction showed a >10% decrease in APP mRNAs containing exon 8 in non-squamous cell lung carcinoma, breast cancer, and colon cancer (as compared to patient-matched controls), indicating a predilection towards APP-751 expression in each of these cancers [58].

The role of the OX-2 domain that is omitted in APP-751 is currently unclear. The OX-2 domain was named for its homology to a region of the OX-2 antigen (an immunoglobulin superfamily member found on neuronal and lymphoid cells), but the function of this specific sequence within the OX-2 protein is not known [59]. Hence, on a mechanistic basis, what effect is exerted by the presence versus absence of exon 8 in cancer cells is not yet apparent. An alternative perspective (proposed by Misquitta-Ali et al. [58]) is that exclusion of the OX-2 domain could alter the production of specific APP cleavage products, and thus the ability of the APP-751 isoform to facilitate tumorigenesis is actually a consequence of the different levels of these products.

## EXPRESSION OF APP FAMILY MEMBERS IN CANCER

APP and/or APLP2 expression is aberrantly altered in many types of cancers (Table 1), such as pancreatic [42, 60, 61], colon [62], breast [63, 64], prostate [65], lung [66], and other cancers [43, 67-81]. Furthermore, APP and APLP2 have been shown to have a range of roles in cancer cells, including both pro-growth and pro-invasion functions. In this review, we will now focus on the expression and functions of APP and APLP2 in specific types of cancer.

### APP family members in germ cell cancers

APLP2 is expressed in normal mouse germ cells, is present on the plasma membrane on mature spermatozoa [82], and has been implicated in sperm survival [83]. Notably, recent studies done by Venkataramani et al. [80], reported that APP expression positively correlates with pluripotency-linked gene expression in testicular germ cell cancers but APLP2 expression does not, indicating that APP (and not APLP2) is a biomarker of pluripotent stem cell transformation. APP is widely expressed at increased levels in several subsets of testicular germ cell tumors, such as seminomas, choriocarcinomas, yolk sac tumors and teratomas [80].

### APP family members in Ewing's sarcoma

In cell lines derived from the pediatric cancer Ewing's sarcoma, APLP2 is typically overexpressed [71]. Upon radiation treatment, increased expression of APLP2 reduces the proportion of Ewing's sarcoma cells in the sub-G1 stage (i.e., the apoptotic subset) [71]. Consistent with APLP2's ability to increase Ewing's sarcoma cell survival following radiation treatment, higher APLP2 overexpression was found in Ewing's sarcoma cells that had resisted lysis by lymphokine-actived killer cells (which destroy target cells by an apoptotic mechanism) [71]. APLP2 overexpression also reduces the level of MHC class I molecules at the plasma membrane of Ewing's sarcoma cell lines [72]. This effect of APLP2 on MHC class I has also been observed with HeLa cells and other cancer cell lines [76, 84-87], suggesting that APLP2 may generally assist in cancer immune escape from T cell killing.

### APP family members in breast cancer

APP expression is increased in breast cancer cell lines that exhibit greater metastatic tendencies, such as motility and proliferation [64]. When APP in a variety of breast cancer cell lines with increasing metastatic potential was knocked down, the cell lines had reduced cell growth and underwent G1 arrest due to induction of the cell cycle inhibitor p27^kip1^ [64]. Additionally, induction of apoptosis markers such as cleaved caspase 3 and the PARP cleavage product were also noted in the breast cancer cell lines in which APLP2 expression had been knocked down, especially in those cell lines that had been identified as having higher metastatic potential [64]. The APP-knockdown breast cancer cells also showed decreased tumor growth in both a 3D *in vitro* cell culture and in an *in vivo* mouse model [64]. APP was also shown to induce migration in breast cancer cells, especially in the presence of IGF-1 [64]. Another study demonstrated that APP was positively correlated with a higher risk of recurrence in ER-positive breast cancer cases, as compared to ER-negative cases, suggesting that increased APP is associated with a worse prognosis [63].

### APP family members in prostate cancer

Studies done by Takayama et al. [88] showed APP induces androgen-mediated signaling pathways contributing to the growth and proliferation of prostate cancer. Also, immunohistology revealed a lack of APP in normal human prostate cells, while in tumor samples from a group of prostate cancer patients with a 50% survival rate there was intense cytoplasmic staining of APP [88]. The importance of APP in prostate cancer was further validated by an *in vivo* animal model in which knock-down of APP repressed tumor growth [88]. In addition, APP has been demonstrated to be involved in the migration and proliferation of prostate cancer cells via mechanisms involving metalloproteinases and epithelial-to-mesenchymal transition-related pathways [65].

### APP family members in lung cancer

APP, especially the secreted form of APP, is upregulated in lung cancers [66]. APP's role in this type of cancer was further verified by a recent study by Sobol et al. [74]. Using APP-specific siRNA transfection of non-small cell lung cancer (NSCLC) cells, this group showed that upon APP downregulation there was destabilization of cyclin C, leading to G0/G1 cell cycle arrest, as well as to decreased phosphorylation of pRb, abnormalities in cell size, and necrosis due to membrane permeabilization [74]. In regard to other APP family members, according to the ONCOMINE database (Compendia Bioscience, Ann Arbor, MI), significant upregulation and downregulation of APLP1 and APLP2 (respectively) was observed in neuroendocrine lung tumors [73]. More work remains to be done, not only to examine the role of APP in lung cancer, but also to investigate the role that APLP1 and APLP2 might play in this particular type of cancer.

### APP family members in melanoma

Metastatic melanoma has a very poor prognosis due to its frequent resistance to traditional chemotherapies and radiation treatments. Botelho et al. [75] showed by immunohistochemistry and immunofluorescence that there is differential expression of transmembrane and secreted APP in the vertical and metastatic growth phase of melanomas, as compared to earlier stages of the disease. Transient knock-down of APP in advanced melanoma cell lines reduced proliferation and increased the expression of melanocyte pigmentation/differentiation markers such as human tyrosinase, tyrosinase-related protein-1, and microphthalmia-associated transcription factor, indicating that the loss of APP leads to a more differentiated phenotype [75]. It was also observed upon APP downregulation in melanoma cells that there was lower expression of ABCB5 (doxorubicin-resistant transporter), which has been implicated in chemoresistance [75]. Consistent with the observed reduction in ABCB5, upon APP downregulation aggressive melanoma cell lines became sensitive to chemotherapeutic drugs to which they were not previously sensitive [75].

### APP family members in pancreatic cancer

Hansel et al. [60] demonstrated that secreted APP enhances cell proliferation in pancreatic cancer cells, as well as thyroid epithelial cells and fibroblasts, by acting as an autocrine growth factor. Other investigators have likewise shown that a secreted form of APP that is produced by α-secretase cleavage aids in cell survival and migration [89]. Pancreatic cancer cell proliferation was significantly reduced upon treatment of the cells with batimastat (which inhibits α-secretase cleavage of APP) along with gemcitabine, as compared to gemcitabine alone [90]. When cells that had been incubated with batimastat were treated with a recombinant form of the secreted APP fragment, growth capacity was restored, confirming that α-secretase-mediated secretion of APP contributes to pancreatic cancer cell growth [90].

Our research group has demonstrated that in addition to overexpression of APP in pancreatic cancer cell lines, there is overexpression of APLP2 (both full-length and cleaved forms) in pancreatic cancer cell lines, and by immunohistochemistry we have demonstrated overexpression of APLP2 in human pancreatic tumor samples [42, 61]. Transient knock-down of APLP2 or APP reduced pancreatic cancer cell growth and viability [42]. We found that a series of cell lines derived from human ductal epithelial cells by transfection with hTERT plus an increasing number of oncogenes had escalating levels of full-length and cleaved APLP2, which suggests that APLP2 may increase gradually during the process of pancreatic cancer development [42].

In an orthotopic mouse model of pancreatic cancer, we demonstrated that down-regulation of APLP2 expression resulted in decreased tumor weight and limited metastasis. We also investigated the expression of APLP2 in human pancreatic cancer metastases, and found that APLP2 is increased in metastatic lesions at many sites, particularly the intestine and the diaphragm [61]. Furthermore, we found positive APLP2 expression in a large proportion (38%) of paired primary tumor and liver metastasis samples from the same patients [61].

### APP family members in colon cancer

Colon cancer also exhibits overexpressed APP and APLP2 [68, 69]. Both *in vitro* and *in vivo* studies have shown that APP promotes growth and proliferation of colon cancer [62]. The gene that encodes APP is part of a genetic signature for increased likelihood of metastasis in patients with early stage mismatch-repair proficient sporadic colon cancer [91].

Consistent with the findings obtained in studies of APP, knock-down of APLP2 reduced proliferation of the Caco2 colon cancer cell line [69]. In colon cancer cells, APLP2 expression is positively correlated with expression of human leukocyte antigen-B-associated transcript 3 (Bat3). Bat3 associates with APLP2 and inhibits its ubiquitylation, thereby blocking its degradation by the proteasome [92]. These findings suggest that Bat3 facilitates the ability of APLP2 to increase colon cancer cell growth by stabilizing APLP2.

## THE REGULATION OF APP AND APLP2

Since it is becoming increasingly clear that APP and APLP2 have pro-cancer functions, how APP and APLP2 expression and function are regulated is also evidently relevant to their roles in cancer. In regard to transcriptional control, there are distinctions between APP and APLP2. The APP promoter has putative recognition sites that are predicted to allow NFĸB/Rel and activator protein-1 (AP-1) to regulate APP expression [93, 94]. The promoter for the APP gene contains transcription factor sites that are absent in the promoter for the APLP2 gene, including a possible heat shock transcription element [95, 96]. Retinoic acid and interleukin-1 have been associated with increased transcription of APP, and retinoic acid also upregulates APLP2 expression [97-99].

The processing of APP and APLP2 is regulated by phosphorylation events. For example, the Pin 1 protein binds to the phosphorylated form of APP at Thr668 [100], and this binding regulates the processing of APP [101]. In addition, the processing of APLP2 is influenced by epidermal growth factor, phorbol 12-myristate 13-acetate (PMA), IGF-1, and retinoic acid [36, 102]. Epidermal growth factor and PMA activate protein kinase C-ε/δ, leading to cleavage and processing of APLP2 via the mitogen-activated protein kinase pathway in corneal epithelial cells [102]. Phosphorylation of protein kinase C by IGF-1 initiates intracellular events leading to the cleavage and shedding of APLP2 from the cell [36]. In cells of the nervous system, the processing and secretion of APP is increased by the presence of okadaic acid, estrogen, or testosterone [103-105].

## CONCLUSIONS

**Figure 5:**
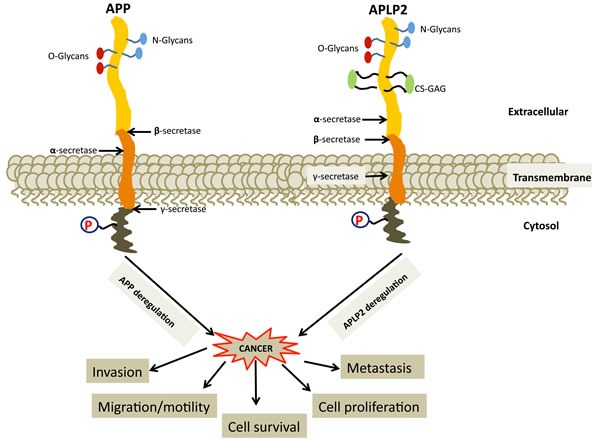
Deregulation of APP and APLP2 causes cancer progression and metastasis, but the roles in cancer of most of the protein interactions involving APP and APLP2 are not well understood Illustrations of transmembrane APP and APLP2 are displayed with cleavage sites indicated. Proteolytic α-, β-. and γ-secretases cleave at various sites on APP and APLP2, generating protein fragments. Interactions between APP and APLP2 with various interacting partners are mediated by glycosylation and phosphorylation.

The APP family members are highly conserved, but despite structural similarities, APP and APLP2 have frequently been observed to have divergent and unique functions. However, both APP and APLP2 are typically upregulated with advancement of cancer progression, and each has been implicated in several phenotypes related to cancer (Table 1; Figure 5). We are also examining the levels within cancer cells of the enzymes (such as beta-secretases) that cleave APP and APLP2, since expression levels of these enzymes regulate the biological influences of both APP and APLP2 [32, 33]. At present, the signaling pathways by which APP and APLP2 wield their effects in cancer cells (which may be multiple pathways) are not well understood, though there are a few clues that the pathways leading to the transcription coactivator YAP (known to be an important factor in cancer growth and migration) might potentially be involved [106-112]. Much remains to be discovered in cancer models about the impact of APP/APLP2 post-translational modifications, such as phosphorylation and glycosylation (Figure 1) [93, 113-125]. Deciphering the specific pathways in which APP and APLP2 function in cancer cells to increase malignancy (and, with APLP2, also to increase cancer immune evasion [76, 84-86]) will be necessary to fully comprehend the roles of these proteins in cancer progression and to develop new therapeutic regimens for cancer that are based on targeting APP and/or APLP2.
